# An overview of the results of hysterosonography prior to *in vitro* fertilization

**DOI:** 10.5935/1518-0557.20170051

**Published:** 2017

**Authors:** Vinicius Medina Lopes, Jean Pierre Barguil, Thaísa Sant'Anna Lacerda, Anna Luíza Moraes Souza, Aluísio Mendes da Rocha Filho, Mariana Fonseca Roller, Eleonora Araújo Barbosa, Natalia Zavattiero Tierno, Joaquim Roberto Costa Lopes

**Affiliations:** 1 Instituto Verhum - Brasília, Distrito Federal-DF, Brazil; 2 Brasília University, DF, Brazil; 3 CENAFERT - Salvador, BA, Brazil

**Keywords:** hysterosonography, hysteroscopy, IVF, endometrial polyp, submucous myomas, uterine synechiae

## Abstract

**Objective:**

This study aimed to analyze the results of hysterosonography performed prior to *in vitro* fertilization (IVF) and to correlate anomalous findings with hysteroscopy.

**Methods:**

Findings from 197 hysterosonograms of patients examined in an assisted reproduction clinic between January 2012 and August 2014 were included. Enrollment criteria: patients in preparation for IVF not recently submitted to uterine examination through hysterosalpingography or hysteroscopy referred to hysterosonography. Uterine cavity evaluation was considered anomalous when one or more of the following were found: polyps, submucous myomas, uterine synechiae, Müllerian duct anomalies. Individuals with cavity abnormalities that might interfere with IVF results were referred to hysteroscopy.

**Results:**

Normal test results were seen in 170/197 of the cases (86.3%). Eighteen of the 197 cases (9.1%) were suspected for polyps, two (1%) for submucous myoma, six (3.5%) for synechiae, and one (0.5%) for Müllerian duct anomalies. Sixteen of the patients diagnosed with abnormalities underwent hysteroscopy to confirm or treat the suspected pathology. In only two cases there was no agreement between tests: one patient suspected for synechiae and another for polyps were not confirmed; another individual suspected for polyps was found to have focal endometrial thickening in hysteroscopy. The positive predictive value (PPV) in our study was 93.7%.

**Conclusion:**

In most cases, the diagnoses obtained by hysterosonography showed normal uterine cavities. The most common anomalous findings were polyps, followed by synechiae, submucous myoma, and Müllerian duct anomalies. Hysterosonography is a good option for evaluating the uterus and offers a high positive predictive value, while hysteroscopy stands as the gold standard.

## INTRODUCTION

Pregnancy rates secondary to *in vitro* fertilization (IVF) treatments relate to embryo implantation capacity and are affected by embryo quality and endometrial receptivity. Uterine diseases and abnormalities such as polyps, myomas, synechiae and congenital malformations may interfere negatively with the embryo implantation process (Cakmak & Taylor, 2011). These endometrial pathologies are very common, particularly in the subfertile population, with prevalence ranging between 11% and 45% (Seshadri *et al*., 2015). Uterine abnormalities were considered the underlying etiology in 10-15% of couples seeking infertility treatment (Gupta *et al*., 2016).

Transvaginal ultrasound (TUS) has been routinely used for decades for uterine evaluation prior to assisted reproductive techniques (ART). However, its diagnostic accuracy is low for the detection of pathologies of the uterine cavity such as polyps, synechiae and submucous myomas (Ragni *et al.*, 2005; Bingol *et al*., 2011). Endometrial and/or uterine abnormalities not detected by TUS are present in 10-30% of infertile and asymptomatic women (Vilela *et al*., 2012). Hysterosonography (HSNG) was developed to improve the diagnosis of these conditions.

HSNG involves the infusion of liquid media in the uterus, such as saline solution, through the endocervical canal, improving uterus visualization. This useful and reliable method for uterine cavity evaluation allows for sensitivity and specificity rates of 98% and 83%, respectively, and positive and negative predictive values of 96% and 91%, respectively (Bingol *et al*., 2011).

In terms of cost, HSNG is two to nine times less expensive than diagnostic hysteroscopy and can replace it in 84% of the cases. According to some authors, hysterosonography in combination with endometrial biopsy, when indicated, may substitute for hysteroscopy as the gold standard for uterine cavity evaluation (Jansen *et al.*, 2006).

HSNG is indicated in the evaluation of the uterine cavity of women with abnormal pre and post-menopausal uterine bleeding, infertility, recurrent pregnancy loss, suspected uterine cavity abnormality, myoma, polyps or synechiae (ACOG, 2012). In addition, HSNG is indicated for focal or diffuse endometrial thickening and ill-defined endometrial images detected by TUS, acting as a complementary methodology (Almog *et al.*, 2011; Yang *et al*., 2013).

HSNG has been recently more used to detect uterine cavity abnormalities prior to ART cycles to improve treatment success rates and decrease the number of cycle cancellations and embryo implantation failures. Some of the factors contributing to the dissemination and acceptance of the technique include the fact that it is less painful, less expensive, less invasive, and requires a shorter learning curve when compared to hysteroscopy (Abou-Salem *et al*., 2010; Bartkowiak *et al*., 2006; Farquhar *et al*., 2003). Thus, it may be indicated in the evaluation of the uterine cavities of women scheduled to undergo IVF treatments (Vilela *et al*., 2012).

There is an ongoing debate about the value of hysteroscopy performed routinely prior to IVF, and conclusive evidence of its benefits is yet to be presented. According to the NICE guidelines, hysteroscopy should not be offered as part of the initial infertility investigation, unless clinically indicated (NICE, 2013). Similarly, the European Society for Human Reproduction and Embryology (ESHRE) guidelines for female infertility limit the use of hysteroscopy to the confirmation and treatment of suspected uterine pathology, but make no reference to using of this technique prior to IVF (ESHRE, 2008). Therefore, human reproduction societies have not manifested yet whether the evaluation of the uterine cavity is needed through either hysteroscopy or HSNG.

This study aimed to analyze the results of hysterosonography tests performed prior to IVF and verify how abnormal findings correlated with hysteroscopy.

## MATERIALS AND METHODS

This retrospective study included the medical records of the patients seen by a physician between January 2012 and August 2014 in an assisted reproduction clinic and all cases in which HSNG was used. Hysterosonography was performed on individuals preparing for IVF who had not undergone uterine cavity evaluation through hysterosalpingography or hysteroscopy for at least a year. Patients submitted to examination for other indications were excluded from the study.

**Figure 1:**
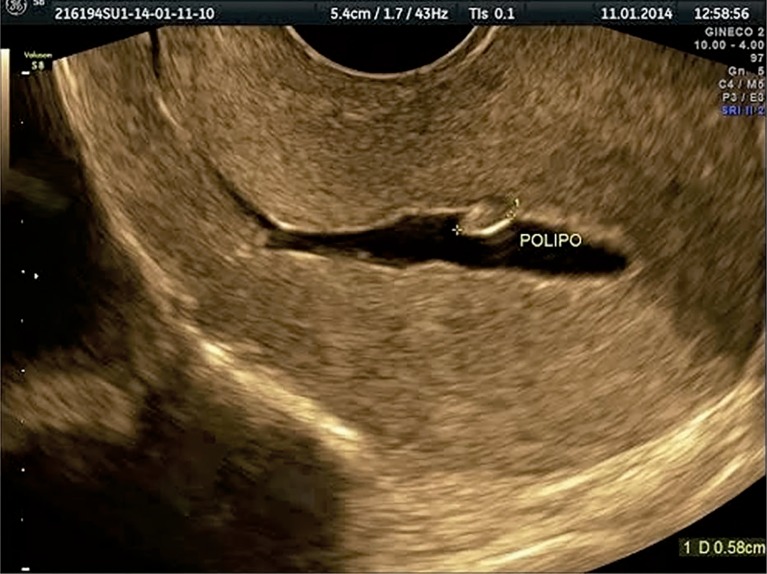
The distention of the uterine cavity produced after the infusion of saline solution allows for better visualization of pathologies. An endometrial polyp measuring 5.8 mm can be seen on the back wall.

The patients underwent HSNG without previous preparation, in the first phase of the cycle, after the end of the menstrual period or in any day when using combined hormonal contraceptive. After speculum placement and visualization of the cervix and external orifice (EO), the cervix was prepped with topical povidone-iodine. Patients allergic to iodine were prepped with an aqueous solution of chlorhexidine. A catheter (Sydney IVF, Cook, Australia) was introduced via the EO of the cervix and positioned in the uterine cavity. The infusion of preheated 0.9% sterile saline solution was then started to distend the cavity under continuous pressure, separating the opposing walls of the endometrium. Using conventional 2D TUS, the anechoic fluid juxtaposed against the echogenic endometrium was visualized, providing a clear image of the uterine lining (Figure 1).

The cavity was deemed abnormal when polyps, submucous myomas, synechiae or Müllerian duct anomalies were detected. The cases suspected for cavity abnormalities that might interfere with the outcome of IVF were referred for hysteroscopy. The test results were then compared to the HSNG findings and the positive predictive value calculated.

## RESULTS

One hundred and ninety-seven patients underwent hysterosonography within the time period comprised in the study. No abnormalities were seen in 170/197 of the cases (86.3%). Examination revealed polyps in 18 cases (9.1%), synechiae in seven (3.5%), submucous myomas in two (1%), and Müllerian duct anomalies in one (0.5%) (Table 1). Twenty-seven patients had abnormal findings, and 16 underwent hysteroscopy to confirm or to treat the suspected pathology. In eleven patients no reports were found or the procedure was performed. Four of these 11 individuals did not return to the service after the prescription of hysteroscopy, and seven underwent IVF without hysteroscopy. Five of these seven patients had polyps smaller than 1 cm; one had an arcuate uterus; and one refused to be tested, although she had a submucous myoma.

**Table 1:** HSNG findings prior to IVF*

HSNG Findings	N (%)
Normal	170 (86.3)
Endometrial Polyps	18 (9.1)
Submucous Myoma	2 (1)
Synechiae	7 (3.5)
Müllerian Duct Anomalies	1 (0.5)

* The Positive Predictive Value was 93.7%.

Disagreement between the two tests was observed in only two patients submitted to hysteroscopy (2/16), one suspected for synechiae and the other for polyps, neither of which confirmed. In this last case, hysteroscopy revealed focal endometrial thickening. The positive predictive value found in our study was 93.7%.

## DISCUSSION

In this study, uterine cavity abnormalities were found in 13.7% of the infertile patients submitted to HSNG prior to IVF. This finding agrees with the results published in another study, in which 13.3% of 60 infertile women analyzed had abnormalities on HSNG (Sitimani *et al*., 2016). However, another study reported abnormalities in 24.8% of 250 infertile women submitted to HSNG (Gupta *et al*., 2016).

The most frequent pathologic finding in HSNG in this study was endometrial polyps (9.1%). IVF was performed in five patients not submitted to hysteroscopy, since small polyps were deemed unable to interfere with treatment outcomes (Check *et al*., 2011). In agreement with our study, other authors also described endometrial polyps as the most prevalent finding (12.5%) (Vilela *et al*., 2012). However, other studies reported that submucous myomas (18.1%) were found more frequently than endometrial polyps (13.6%) (Gupta *et al*., 2016).

Other authors reported lower incidences of uterine cavity disorders, with endometrial polyps and myomas seen in 5% and synechiae in 1.7% of patients (Sitimani *et al.*, 2016). A study enrolled 241 infertile women to evaluate the presence of endometrial polyps. The patients were submitted to HSNG and hysteroscopy, and the sensitivity, specificity, accuracy and error in the detection of endometrial polyps by HSNG were 97.3%, 95.8%, 96.2%, and 3.7%, respectively (Radwan *et al*., 2014). Positive and negative predictive values were 91.1% and 98.7%, respectively. The agreement between HSNG and hysteroscopy combined with histopathology examination was very high, indicating that hysterosonography is a safe and highly sensitive and specific method to diagnose endometrial polyps. In our study, all but one of the women suspected for endometrial polyps submitted to hysteroscopy had their diagnoses confirmed.

Intrauterine synechiae were the second most common finding in our study (3.5%). This percentage is similar to what is found in the literature, with values ranging between 1.7% and 2% (Gupta *et al*., 2016; Sitimani *et al*., 2016). It should be noted that in the present study four of the six cases diagnosed with synechiae by HSNG and confirmed with hysteroscopy had a history of post-abortion curettage. The other two had a history of postpartum curettage and foreign body excision. The six patients suspected for synechiae were referred to hysteroscopy, since hysteroscopic repair of the lesion provides improved reproductive results (Taylor & Gomel, 2008).

Only 1% of the hysterosonograms revealed the presence of submucous myomas, yielding a lower prevalence than the rates reported in literature, which range between 5% and 18% (Gupta *et al*., 2016; Sitimani *et al*., 2016; Vilela *et al*., 2012). When ultrasound examination found unequivocal evidence of a submucous component in the myomas, the patients were referred directly to hysteroscopy. This fact may explain the lower incidence of submucous myomas diagnosed by HSNG in our study. According to Gupta *et al*. (2016), these myomas are the second cause of uterine cavity abnormality (6.8%). TUS, HSNG, and hysteroscopy had sensitivities of 58.8%, 82.8%, and 76.4%, and specificities of 96.7%, 90.3%, and 90.16%, respectively, in detecting this disorder.

A comparative study between HSNG, TUS, and hysteroscopy was performed with 98 infertile patients scheduled to undergo IVF to assess the use of HSNG as a method to diagnose intracavitary uterine pathologies in infertile patients. HSNG yielded a sensitivity of 98%, a specificity of 94%, a PPV of 95%, and a NPV of 98% when compared to hysteroscopy. The accuracy of HSNG was significantly better than ultrasonography for intracavitary pathologies and uterine polyps.

In this study, only two cases had no agreement between HSNG and hysteroscopy. A patient suspected for endometrial polyps was found to have focal endometrial thickening on hysteroscopy. Therefore, only one patient suspected for an anomaly on HSNG did not have an anomaly, yielding a positive predictive value of 93.7%.

A systematic review and meta-analysis performed on the accuracy of HSNG to diagnose cavity abnormalities prior to ART showed 88% sensitivity and 94% specificity in the detection of endometrial abnormalities. The authors concluded that this is a highly sensitive diagnostic method comparable to the gold standard, hysteroscopy, in the detection of intrauterine abnormalities in subfertile women (Seshadri *et al*., 2015). It is a highly sensitive and specific test in the diagnosis of uterine polyps, submucous myomas, uterine anomalies, and synechiae, which may be used as a screening tool for subfertile patients prior to IVF. Therefore, in the present study, hysteroscopy was not performed in patients with normal HSNG findings because we considered that there would be no benefit for them in doing so. Thus, it was not possible to calculate the sensitivity, specificity, or negative predictive value, since hysteroscopy was only performed in patients suspected for abnormalities in HSNG.

## CONCLUSION

The uterine cavities of most patients assessed by hysterosonography prior to IVF were normal. The most common anomalous findings were polyps, followed by synechiae, submucous myoma, and Müllerian duct anomalies. Most of these findings might compromise the outcomes of IVF procedures. Hysterosonography is a good option for evaluating the uterus with a positive predictive value of 93.7% in this sample, while hysteroscopy stands as the gold standard.
